# Highly Effective Self-Propagating Synthesis of Lamellar ZnO-Decorated MnO_2_ Nanocrystals with Improved Supercapacitive Performance

**DOI:** 10.3390/nano11071680

**Published:** 2021-06-25

**Authors:** Luming Li, Jing Li, Hongmei Li, Li Lan, Jie Deng

**Affiliations:** 1 School of Food and Biological Engineering, Chengdu University, Chengdu 610106, China; liluming@cdu.edu.cn (L.L.); lihongmei@cdu.edu.cn (H.L.); 1020200502@jxstnu.edu.cn (L.L.); 2 School of Chemical Engineering, Sichuan University, Chengdu 610065, China

**Keywords:** energy storage, MnO_2_, dopant, nanocrystals, self-propagating

## Abstract

A series of MO*_x_* (M = Co, Ni, Zn, Ce)-modified lamellar MnO_2_ electrode materials were controllably synthesized with a superfast self-propagating technology and their electrochemical practicability was evaluated using a three-electrode system. The results demonstrated that the specific capacitance varied with the heteroatom type as well as the doping level. The low ZnO doping level was more beneficial for improving electrical conductivity and structural stability, and Mn10Zn hybrid nanocrystals exhibited a high specific capacitance of 175.3 F·g^−1^ and capacitance retention of 96.9% after 2000 cycles at constant current of 0.2 A·g^−1^. Moreover, XRD, SEM, and XPS characterizations confirmed that a small part of the heteroatoms entered the framework to cause lattice distortion of MnO_2_, while the rest dispersed uniformly on the surface of the carrier to form an interfacial collaborative effect. All of them induced enhanced electrical conductivity and electrochemical properties. Thus, the current work provides an ultrafast route for development of high-performance pseudocapacitive energy storage nanomaterials.

## 1. Introduction

The energy storage of pseudocapacitors is based on both ion adsorption and fast surface redox reactions, which can beneficially endow high specific capacitance and energy density compared to electrochemical double-layer capacitors [1,2]. The transition metal oxides, such as MnO*_x_*, RuO_2,_ CoO*_x_*, Cr_2_O_3_, CuO, NiO, CeO_2_, and FeO*_x_*, are widely employed, thanks to their low cost, low toxicity, and environmental friendliness, as active electrode materials for pseudocapacitors [3,4,5,6]. For example, manganese oxide (MnO_2_) has stood out because of its amazingly high theoretical specific capacitance of 1370 F·g^−1^ [7]. However, poor inherent electronic conductivity (10^−5^–10^−6^ S/cm) usually imparts the bulk of MnO_2_ materials with low practical capacitances (less than 100 F·g^−1^), well below the theoretical value [8]. This severely hampers the practical delivery of MnO_2_ as high-performance pseudocapacitive electrode nanomaterials.

To dispose of this problem, MnO_2_-based composites combined with conductive materials, such as carbon materials, polymers, metals, and some transition metal oxides, have attracted much attention [9,10]. However, the promotion of MnO_2_ conductivity through external conductive improvements is very limited, due to the weak interactions of the MnO_2_/conductor interfaces. The question of how to efficiently integrate nanostructured MnO_2_ and a conductive modifier using a facile route to enhance pseudocapacitance performance of energy storage devices, as well as cycle stability, is of significance and has attracted much attention. Chen et al. reported that the NiO@MnO_2_ core/shell nanocomposites prepared with a two-step method resulted in improved electronic conductivity and enhanced specific capacitance, with an excellent cycling stability of 81.7% retention after 2000 cycles at a current density of 1 A·g^−1^ [11]. Lu et al. found that MnO_2_ loaded on hydrogen-treated TiO_2_ (H-TiO_2_) conducting nanowire (H-TiO_2_@MnO_2_ NWS) electrodes could deliver a high specific capacitance with a cycling performance of 91.2% [12]. Zhu et al. developed a simple self-assembly route and prepared a CeO_2_@MnO_2_ heterojunction nanostructure with an excellent capacitive performance thanks to the synergistic effect between CeO_2_ and MnO_2_ [13]. In addition, the doping of hetero elements, such as Co, Ce, Cu, and Zn, can also validly vary the electronic structure of MnO_2_ and thereby induce better electronic conductivity and electrochemical performance, as well as improved retention ability [7,14,15]. Thus, it is highly desirable to fabricate heteroatom-modified MnO_2_ hybrid materials with special constructed nanostructures and controlled crystal morphologies via a facile step.

In this study, the enhanced conductivity of MnO_2_-based composites was treated as a classical factor in order to command their electrochemical performance via regulation of doping elements and optimization of dopant levels, as well as bridging of the special nanostructure. According to our previous studies, hydrocarbons and CO decomposed by copper acetate can be easily ignited in oxygen-enriched circumstances of potassium permanganate after their mixed grinding, leading to formation of novel CuO-δ-MnO_2_ hybrid mixtures [16]. This sample provides catalytic activity comparable to that of the Pt-based catalyst for toluene catalytic combustion, owing to the synergetic effect of combining MnO_2_ and additional copper oxide [16]. Inspired by the foregoing synergies between CuO and MnO_2_, we hypothesized that incorporating ZnO into the optimized lamellar MnO_2_ systems might produce functionalized electronic structures for promoting intrinsic conductivity and augmenting electrochemical performance. In this study, a series of metal oxide-functionalized MnO_2_ electrode materials were prepared by a superfast and eco-friendly self-propagating technology (SPT), and an advisable amount of Zn-decorated lamellar MnO_2_ nanocrystals were found to deliver excellent electrochemical performance and high capacitance retention. These outcomes have significant potential for engineering state-of-the-art doped MnO_2_ nanostructures for electrochemical energy storage.

## 2. Materials and Methods

### 2.1. Preparation of Layered MO_*x*_-δ-MnO_2_

**Figure 1:**
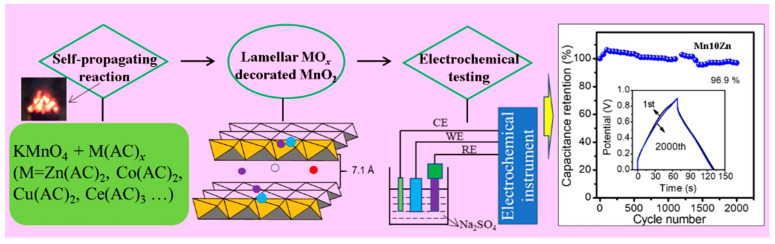
Schematic illustration of the self-propagating technology (SPT) strategy and electrochemical performance evaluation.

Layered MO*_x_*-δ-MnO_2_ (M = Co, Ni, Zn, Ce) electrode materials were fabricated through a superfast SPT process with potassium permanganate (KMnO_4_) and acetic salt (M(CH_3_COO)_x_·*x*H_2_O, where M was labeled as Co, Ni, Zn, Ce) (Figure 1). Specifically, M(CH_3_COO)_2_·*x*H_2_O was mixed with KMnO_4_ in a mortar at a fixed molar ratio of Mn to M (8:1), and the mixture was ground to homogeneity for approximately 10 min. Then, the mixture was placed on a smooth stainless plate and ignited with a flame for several seconds (3–5 s). After that, the final cooled black product was washed until the pH value was 7, then it was filtered and dried at 100 °C for 12 h. The related produced catalysts were marked by Mn8Co, Mn8Ni, Mn8Zn, and Mn8Ce. The series of ZnO-δ-MnO_2_ catalysts with different zinc oxide contents were also synthesized with the above strategy with KMnO_4_ and (CH_3_COO)_2_·Zn. The molar ratios of Mn/Zn were adjusted to 5:1 and 10:1, and the obtained samples were listed as Mn5Zn and Mn10Zn, respectively.

### 2.2. Characterization of Synthesized Materials

X-ray diffraction (XRD) was implemented on a Haoyuan DX-2700 with Cu Kα (Haoyuan Co., Liaoning, China). Scanning electron microscopy (SEM) was also carried out on a FEI Nova NanoSEM 450 microscope (FEI, Hillsboro, OR, USA). N_2_ adsorption–desorption isotherms were carried out using a V-Sorb 2800P analyzer (Gold APP Instruments Corporation, Beijing, China). An inductively coupled plasma mass spectrometer (Thermo Scientific, ICP-MS, Waltham, MA, USA) equipped with an automatic sampler (ASX-560) was employed to investigate the actual molar ratio of Mn/Zn. The surface species were examined by X-ray photoelectron spectroscopy (XPS, XSAM800) (KRATOS, Manchester, UK).

### 2.3. Electrochemical Tests

The electrochemical tests were performed on a CHI 660E workstation (Chenhua Instrument Co., Shanghai, China) using a three-electrode system with a Pt mesh as the counter electrode (CE) and a saturated Hg/HgO electrode as the reference electrode (RE), as depicted in Figure 1. It can be noted that the electrode of the layered MO*_x_*-δ-MnO_2_ materials was manufactured by mixing the conductive agent of black carbon and polyvinylidene fluoride, in which the mass ratio was designated as 8:1:1 in *N*-methyl-2-pyrrolidone (NMP). The mixture was ground and loaded on nickel foam (1 cm × 1 cm) with roughly 1 mg of layered MO*_x_*-δ-MnO_2_ materials. Then, 1 M Na_2_SO_4_ aqueous solution was employed as an electrolyte for all the electrochemical measurements, due to its environmental friendliness, cost-effectiveness, and electrochemical stability.

## 3. Results

**Figure 2:**
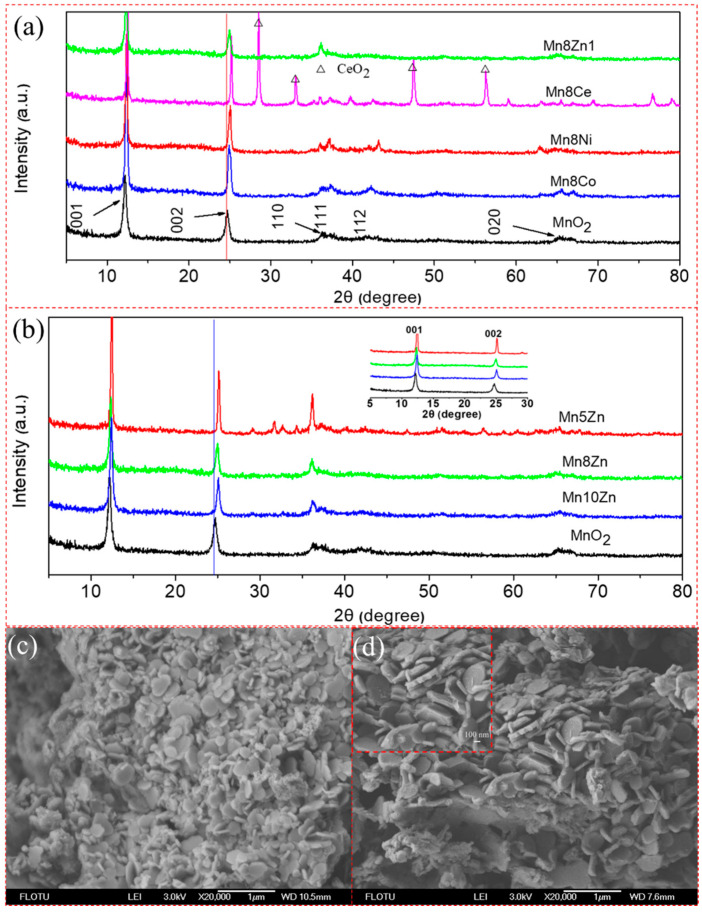
(**a**,**b**) XRD patterns of the layered MO*_x_*-δ-MnO_2_ (M = Co, Ni, Zn, Ce) materials; (**c**,**d**) SEM images of the Mn5Zn and Mn10Zn samples, respectively. Inserted image of (**b**) is the local enlarged XRD patterns ranges from 2θ = 5 to 30 degree.

The phase composition and morphology of the synthesized layered MO*_x_*-δ-MnO_2_ (M = Co, Ni, Zn, Ce) materials were studied by XRD and SEM (Figure 2). It can be observed that the MO*_x_*-δ-MnO_2_ (M = Co, Ni, Zn) samples with the same doping levels featured the typical characteristic diffraction peaks at the 2θ values of 12.3 and 24.9° (Figure 2a), which can be attributed to the crystal planes of (001) and (002) of the parent lamellar MnO_2_ (JCPDS no. 43-1456) [17]. Moreover, the diffraction peak slightly shifted to a higher location with the higher dopant (Zn) level (Figure 2b and, inserted, an enlarged view), which can be ascribed to the heteroatom-induced lattice distortion of parent manganese oxide [16]. It is worth noting that the doping of cerium oxide probably resulted in a higher degree of lattice distortion, along with a more complex crystal composition, because of the large ion radius of Ce and the formation of a fluorite structure of CeO_2_ (Figure 2a).

Figure 2c,d display the SEM images of the Mn5Zn and Mn10Zn samples, respectively. It can be seen that both had lamellar morphologies, and the high molar ratio of Mn/Zn brought about a thick lamellar structure. It was suggested that overloading with ZnO might restrain the formation of high specific surface areas in the modified δ-MnO_2_ catalysts during the SPT process, as the N_2_ adsorption–desorption curves showed that the value (33.2 m^2^/g) of the specific surface area of Mn10Zn was higher than that of Mn5Zn (22.8 m^2^/g) (Figure S1). This corresponds well with our previous report [16]. To investigate the dispersion of elements of the Zn-decorated MnO_2_, energy-dispersive spectroscopy (EDS) was also employed in this study. Figure S2 depicts the dispersion of elements of Mn, Zn, and O, respectively. It was found that a lot of green points of Zn nanoparticles were highly dispersed on MnO_2_ nanosheets, indicating the uniform dispersion of ZnO nanoparticles (NPs) on the birnessite-type MnO_2_ carrier.

**Figure 3:**
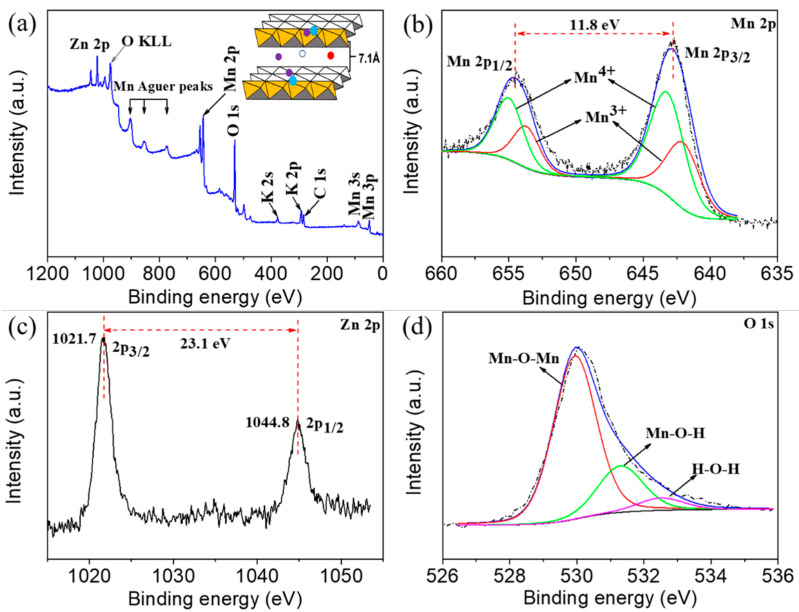
XPS spectra of the Mn10Zn sample: (**a**) survey scan, (**b**) Mn 2p, (**c**) Zn 2p, and (**d**) O 1s.

Figure 3a presents the XPS survey spectrum of the Mn10Zn sample. Mn (642 eV), O (530 eV), K (292 eV), and Zn (1022 eV) can be observed based on the binding energy values. According to previous reports, K^+^ has a positive effect on improving the conductivity and stabilizing the structure of MnO_2_ [18,19]. In our recent report, we also found that the moderating effect of potassium ions remained within the structure of the mezzanine of the δ-MnO_2_ catalysts and probably played a key role in adjusting the structure stability due to the pillared effect of K^+^ [20]. Figure 3b shows that the high-resolution Mn 2p spectrum consisted of two peaks at 642.1 eV (Mn 2p3/2) and 653.9 eV (Mn 2p1/2), with a spin-energy spilt-up of 11.8 eV, which is in good agreement with previous studies from the literature and thus evidences the presence of MnO_2_ [21]. In addition, the molar ratio of Mn^4+^/Mn^3+^ was roughly 1.7 from the integration of the correlating peak areas, implying that the Mn^4+^ ion was the main component for Mn10Zn. The Zn 2p_3/2_ and Zn 2p_1/2_ profiles were observed at the locations of 1021.7 eV and 1044.8 eV, where the value of energy separation was 23.1 eV. This result directly confirmed the existence of ZnO and/or interfacial ZnMn_2_O_4_ (Figure 3c) [22]. Moreover, the local environment of oxygen played an important role in regulating the electrochemical performance. There were three bonding states of oxygen, including Mn-O-Mn (529.8 eV), Mn-O-H (531.3 eV), and H-O-H (532.6 eV), and the level of Mn-O-Mn was dominant, as displayed in Figure 3d.

**Figure 4:**
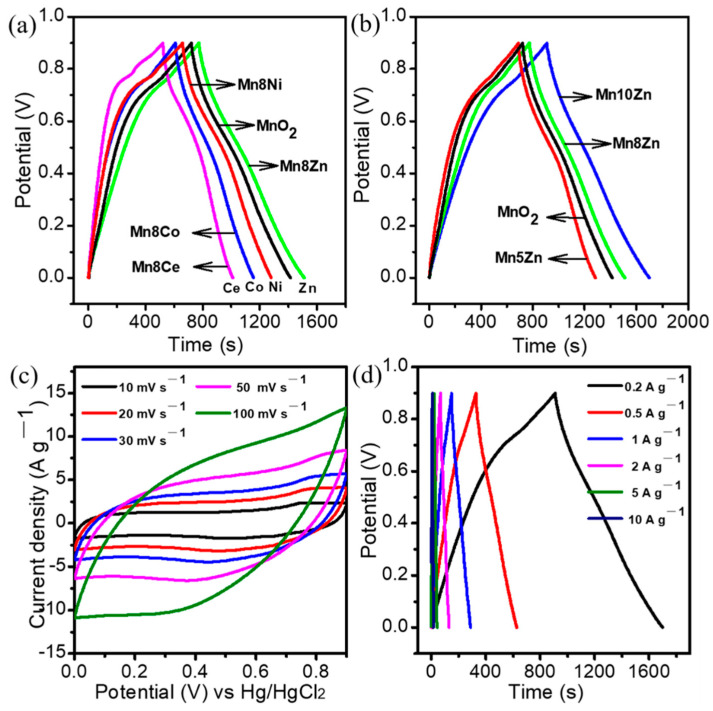
(**a**,**b**) GCD profiles of the layered MO*_x_*-δ-MnO_2_ (M = Co, Ni, Zn, Ce) materials; (**c**,**d**) CV and GCD patterns of Mn10Zn.

The value of the specific capacitance for electrode materials reflects the prospect of practical applicability to some extent. In this study, we employed galvanostatic charge/discharge (GCD), cyclic voltammetry (CV), and impedance experiments to assess the electrochemical application of layered MO*_x_*-δ-MnO_2_ (M = Co, Ni, Zn, Ce) bimetallic materials in 1 M Na_2_SO_4_ aqueous solution. Figure 4a,b provide the GCD profiles of layered MO_*x*_-δ-MnO_2_ with the same Mn/M ratio of 8 and of the lamellar Zn-decorated MnO_2_ nanocrystal with a different doping level, respectively. It was found that zinc doping with a Mn/Zn ratio of 8 resulted in the best improvement of the specific capacitance and a higher value 163.6 F/g compared to the parent MnO_2_ (153.7 F/g) and other heteroatomic modulations, while the Mn8Ce afforded a much-diminished specific capacitance of 108.6 F/g, manifesting the negative doping effect. It can be noted that the improved electrochemical performance of CeO_2_ mainly hinged on the specific surface area, particle size, morphology, and defect states [23]. In this study, CeO_2_ layer-doped MnO_2_ electrode materials were prepared using a superfast SPT process within several seconds, and it was a little hard to efficiently control the vital parameters of their structures. This was most likely a result of the negative enhancement effect of the modifier. However, the specific capacitance of Zn-decorated MnO_2_ could be further promoted by adjusting the doping level of Zn; for example, the value of Mn10Zn was increased up to 175.3 F/g. These data clearly indicate that the boost of electrochemical performance of MnO_2_ strongly hinged on the dopant type and the optimized content. Qiao et al. found that a suitable amount of ZnO with an ionic conductor of La/Pr co-doped with CeO_2_ can lead to increasing the power density in solid oxide fuel cells, but further increases of ZnO to 40–60 wt% only brought about a negative impact on power density [24]. Similarly, overloading of ZnO with the sample of Mn/Zn of ratio 5:1 also provided a negative impact on the specific performance, which corresponds well with previous studies from the literature. It is well-known that incorporating appropriate atoms or ions into host lattices of MnO_2_ can enhance its electronic conductivity and electrochemical performance [17]. Herein, the enhanced electrochemical capacitance should have been due to the synergistic effect from the entry of heteroatoms into the framework of MnO_2_ and interfacial interaction between the ZnO nanocatalysts and the parent MnO_2_ carrier.

**Figure 5:**
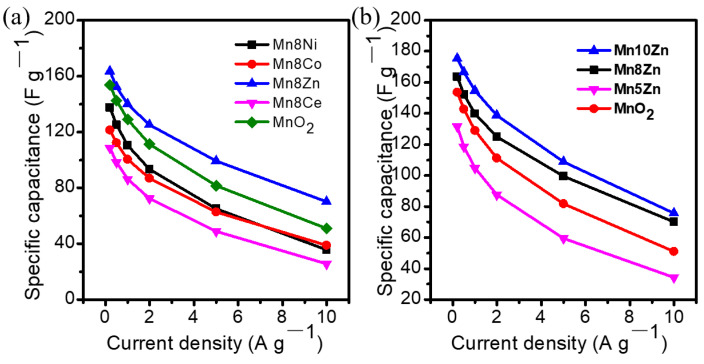
(**a**,**b**) The specific performance of the layered MO*_x_*-δ-MnO_2_ (M = Co, Ni, Zn, Ce) materials at different current densities.

Additionally, the CV of Mn10Zn exhibited a typical rectangle shape within a potential window of 0–0.9 V at different scan rates (Figure 4c), revealing that the reversible redox reaction rapidly occurred at the interface of Mn10Zn and the electrolyte ion via the following reaction mechanism (Equation (1)), suggesting good pseudocapacitive behavior [25]. Moreover, GCD curves displayed symmetrical charge–discharge behavior at different current densities, which also implied a fast and reversible Faradic reaction between the alkali cation of Na^+^ and the lamellar ZnO-decorated MnO_2_ nanocrystals (Figure 4d). It is worth noting that the specific capacitance for all the samples decreased with the increase of current density owing to the inadequate reaction between the active materials and electrolyte ions under fast-changing potential [26]. Figure 5a shows the sequence of electrochemical rate capabilities with the increase of current density as follows: Mn8Zn (42.8%), Mn8Co (32.1%), Mn8Ni (25.9%), and Mn8Ce (23.6%). This was consistent with the variation trend of the specific capacitance at the same current density. Moreover, the relatively low content of ZnO doping modification led to a better rate performance, as shown in Figure 5b. However, the Zn-decorated lamellar MnO_2_ nanocrystal with optimized doping level (Mn10Zn) possessed a high-performance rate (43.1%) compared to the other heteroatom-doped MnO_2_ (Figure 4d and Figure 5b), and the specific capacitance remained 74.8 F/g at a high charge/discharge rate of 10 A g^−1^.

(1) $${\left(\mathrm{Mn}10\mathrm{Zn}\right)}_{\mathrm{surface}}+{\mathrm{Na}}^{+}+e^{-}⟷{\left[{\left(\mathrm{Mn}10\mathrm{Zn}\right)}_{\mathrm{surface}}\right]}^{-}{\mathrm{Na}}^{+}$$

**Figure 6:**
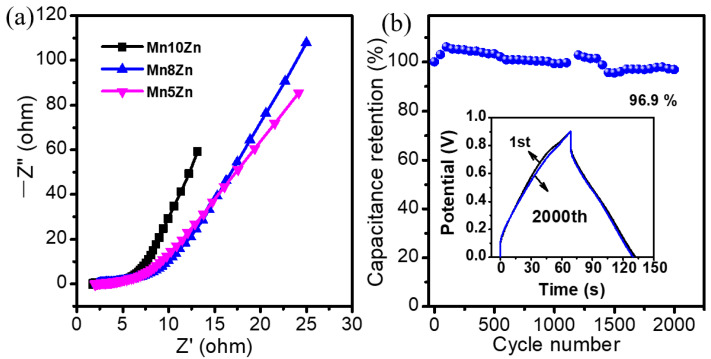
(**a**) Nyquist plot of the impedance of Zn-doped MnO_2_ materials; (**b**) cyclic performance of Mn10Zn at a constant current of 0.2 A·g^−1^, the inserted image of (**b**) is the first and 2000th GCD patterns of Mn10Zn.

Moreover, the slope of the Warburg resistance of the Zn-decorated MnO_2_ nanocatalysts with low-level modification was higher than that with high doping content (Figure 6a), suggesting the decline of charge transfer resistance arising from the optimized surface structure of the MnO_2_ layer. This could account for the negative effect of overdoping modification of heteroatoms. Furthermore, cyclic stability is an important index to evaluate the practical potential of electrode materials. As displayed in Figure 6b, Mn10Zn presented excellent operation stability with a capacitance retention of 96.9% after 2000 cycles at a constant current density of 0.2 A·g^−1^. Therefore, the electrochemical performance of MO*_x_* (M = Co, Ni, Zn, Ce)-doped δ-MnO_2_ electrode materials was closely related to the Mn/Zn molar ratio. Relatively low levels of ZnO modification were conducive to better electrochemical activity for energy storage devices due to the better electronic conductivity, the highly dispersed ZnO phase with a small grain size, and the rich content of the interfacial defects.

## 4. Conclusions

In summary, a series of MO*_x_* (M = Co, Ni, Zn, Ce)-doped δ-MnO_2_ nanosheets were prepared using a superfast and eco-friendly SPT strategy. XRD, SEM, and XPS characterizations confirmed that a small part of the heteroatoms entered the framework and caused lattice distortion of MnO_2_, while the rest of them dispersed uniformly on the surface of the carrier to form an interfacial collaborative effect. All of them induced enhanced electrical conductivity and electrochemical properties. Among the synthesized electrode materials, Mn10Zn hybrid nanocrystals exhibited a high specific capacitance of 175.3 F·g^−1^ and a capacitance retention of 96.9% after 2000 cycles at a constant current density of 0.2 A·g^−1^. This study demonstrates that both the distortion defects of the matrix lattice and interfacial interaction between the dopant and carrier lead to positive effects on electrochemical performances.

## Data Availability

The data can be available upon request from the authors.

## Supplementary Materials

The following are available online at https://www.mdpi.com/article/10.3390/nano11071680/s1. Figure S1. The N_2_ adsorption-desorption curves of Mn_10_Zn and Mn_5_Zn samples. Figure S2. Images of element mapping of Mn, Zn and O, respectively.

## References

[1] Huang Z.H., Song Y., Feng D.Y., Sun Z., Sun X.Q., Liu X.X. (2018). High Mass Loading MnO_2_ with Hierarchical Nanostructures for Supercapacitors. ACS Nano.

[2] Gao P., Metz P., Hey T., Gong Y.X., Liu D.W., Edwards D.D., Howe J.Y., Huang R., Misture S.T. (2017). The Critical Role of Point Defects in Improving the Specific Capacitance of δ-MnO_2_ Nanosheets. Nat. Commun..

[3] Hussain K., Ali I., Hasnain S., Hussain S.S., Hussain B., Khan M.S., Ammar S.M., Hussain B., Hussain A., Javed M.A. (2020). Reagents Assisted Mg-doped CeO_2_ for High-performance Energy-storage Applications. J. Electroanal. Chem..

[4] Subalakshmi P., Ganesan M., Sivashanmugam A. (2017). Synthesis of 3D Architecture CuO Micro Balls and Nano Hexagons and its Electrochemical Capacitive Behavior. Mater. Design.

[5] Bounor B., Asbani B., Douard C., Favier F., Brousse T., Lethien C. (2021). On Chip MnO_2_-based 3D Micro-supercapacitors with Ultra-high Areal Energy Density. Energy Storage Mater..

[6] Wang Y., Guo J., Wang T.F., Shao J.F., Wang D., Yang Y.W. (2015). Mesoporous Transition Metal Oxides for Supercapacitors. Nanomaterials.

[7] Tang C.L., Wei X., Jiang Y.M., Wu X.Y., Han L.N., Wang K.X., Chen J.S. (2015). Cobalt-Doped MnO_2_ Hierarchical Yolk Shell Spheres with Improved Supercapacitive Performance. J. Phys. Chem. C.

[8] Nasser R., Zhang G.F., Song J.M. (2020). Facile and Low-cost Synthesis of Cobalt-doped MnO_2_ Decorated with Graphene Oxide for High Performance 2.3V Aqueous Asymmetric Supercapacitors. Electrochim. Acta.

[9] Kang J., Hirata A., Kang L., Zhang X., Hou Y., Chen L., Li C., Fujita T., Akagi K., Chen M. (2013). Enhanced Supercapacitor Performance of MnO_2_ by Atomic Doping. Angew. Chem. Int. Ed..

[10] Wang J., Tian L., Xie W.L., Wang X., Long X., Sun K., Emin A., Liu D.Q., Fu Y.J., Chen Q. (2020). A Hierarchical Interconnected Nanosheet Structure of Porous δ-MnO_2_ on Graphite Paper as Cathode with a Broad Potential Window for NaNO_3_ Aqueous Electrolyte Supercapacitors. ACS Appl. Energy Mater..

[11] Chen J.J., Huang Y., Li C., Chen X.F., Zhang X. (2016). Synthesis of NiO@MnO_2_ Core/shell Nanocomposites for Supercapacitor Application. Appl. Surf. Sci..

[12] Lu X.H., Yu M.H., Wang G.M., Zhai T., Xie S.L., Ling Y.C., Tong Y.X., Li Y. (2013). H-TiO_2_@MnO_2_//H-TiO_2_@C Core-shell Nanowires for High Performance and Flexible Asymmetric Supercapacitors. Adv. Mater..

[13] Zhu S.J., Jia J.Q., Wang T., Zhao D., Yang J., Dong F., Shang Z.G., Zhang Y.X. (2015). Rational Design of Octahedron and Nanowire CeO_2_@MnO_2_ Core-shell Heterostructures with Outstanding Rate Capability for Asymmetric Supercapacitors. Chem. Commun..

[14] Chen K.F., Pan W., Xue D.F. (2016). Phase Transformation of Ce^3+^-Doped MnO_2_ for Pseudocapacitive Electrode Materials. J. Phy. Chem. C.

[15] Peng R.C., Wu N., Zheng Y., Huang Y.B., Luo Y.B., Yu P., Zhuang L. (2016). Large-Scale Synthesis of Metal-Ion-Doped Manganese Dioxide for Enhanced Electrochemical Performance. ACS Appl. Mater. Inter..

[16] Li L.M., Luo J.J., Liu Y.F., Jing F.L., Su D.S., Chu W. (2017). Self-Propagated Flaming Synthesis of Highly Active Layered CuO-δ-MnO_2_ Hybrid Composites for Catalytic Total Oxidation of Toluene Pollutant. ACS Appl Mater Interfaces.

[17] Yan L.J., Niu L.Y., Shen C., Zhang Z.K., Lin J.H., Shen F.Y., Gong Y.Y., Li C., Liu X.J., Xu S.Q. (2019). Modulating the Electronic Structure and Pseudocapacitance of δ-MnO_2_ Through Transitional Metal M (M = Fe, Co and Ni) Doping. Electrochim. Acta.

[18] Xu N.N., Nie Q., Luo Y.Q., Yao C.Z., Gong Q.J., Liu Y.Y., Zhou X.D., Qiao J.L. (2019). Controllable Hortensia-like MnO_2_ Synergized with Carbon Nanotubes as an Efficient Electrocatalyst for Long-Term Metal-Air Batteries. ACS Appl. Mater. Interfaces.

[19] Yuan Y.F., Zhang C., He K., Chen H.R., Yao W.T., Sharifi-Asl S., Song B.A., Yang Z.Z., Nie A.M., Luo X.Y. (2016). The Influence of Large Cations on the Electrochemical Properties of Tunnel-structured Metal Oxides. Nat. Commun..

[20] Li L.M., Chu W., Liu Y. (2021). Insights into Key Parameters of MnO_2_ Catalyst toward High Catalytic Combustion Performance. J. Mater. Sci..

[21] Bose N., Sundararajan V., Prasankumar T., Jose S.P. (2020). α-MnO_2_ Coated Anion Intercalated Carbon Nanowires: A High Rate Capability Electrode Material for Supercapacitors. Mater. Lett..

[22] Radhamani A.V., Shareef K.M., Rao M.S.R. (2016). ZnO@MnO_2_ Core-Shell Nanofiber Cathodes for High Performance Asymmetric Supercapacitors. ACS Appl. Mater. Interfaces.

[23] Kumar M., Yun J.H., Vishwa B., Singh B., Kim J., Kim J.S., Kim B.S., Lee C.Y. (2018). Role of Ce^3+^ Valence State and Surface Oxygen Vacancies on Enhanced Electrochemical Performance of Single Step Solvothermally Synthesized CeO_2_ Nanoparticles. Electrochim. Acta.

[24] Qiao Z., Xia C., Cai Y.X., Afzal M., Wang H., Qiao J.L., Zhu B. (2018). Electrochemical and Electrical Properties of Doped CeO_2_-ZnO Composite for Low-temperature Solid Oxide Fuel Cell Applications. J. Power Sources.

[25] Zhu S.J., Li L., Liu J.B., Wang H.T., Wang T., Zhang Y.X., Zhang L.L., Ruoff R.S., Dong F. (2018). Structural Directed Growth of Ultrathin Parallel Birnessite on β-MnO_2_ for High-Performance Asymmetric Supercapacitors. ACS Nano.

[26] Zhang M., Chen Y., Yang D., Li J. (2020). High Performance MnO_2_ Supercapacitor Material Prepared by Modified Electrodeposition Method with Different Electrodeposition Voltages. J. Energy Storage.

