# Supplementary material for: Highly Effective Self-Propagating Synthesis of Lamellar ZnO-Decorated MnO2 Nanocrystals with Improved Supercapacitive Performance
Source: Nanomaterials (Basel). 2021 Jun 25;11(7):1680. doi: 10.3390/nano11071680 (PMC8306293; doi:10.3390/nano11071680)
Supplement: Supplementary file 1 [file nanomaterials-11-01680-s001.zip › nanomaterials-1236865-supplementary.pdf]

nanomaterials

Supplementary Materials

Highly Effective Self-Propagating Synthesis of Lamellar ZnO-Decorated MnO_2 Nanocrystals with Improved Supercapacitive Performance

Luming Li [1,2], Jing Li [2,*], Hongmei Li [1], Li Lan [1] and Jie Deng [1,*]

[1] School of Food and Biological Engineering, Chengdu University, Chengdu 610106, China; liluming@cdu.edu.cn (L.L.); lihongmei@cdu.edu.cn (H.L.); 1020200502@jxstnu.edu.cn (L.L.)
[2] School of Chemical Engineering, Sichuan University, Chengdu 610065, China
* Correspondence: jingli0726@g.ucla.edu (J.L.); dengjie@cdu.edu.cn (J.D.)

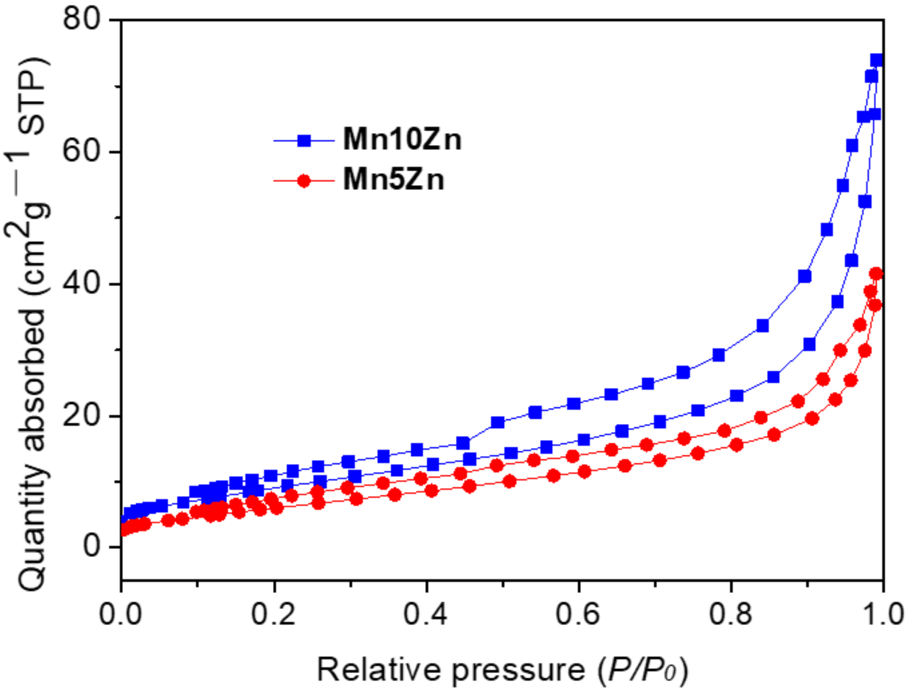

Figure S1. The N_2 adsorption-desorption curves of Mn10Zn and Mn5Zn samples.

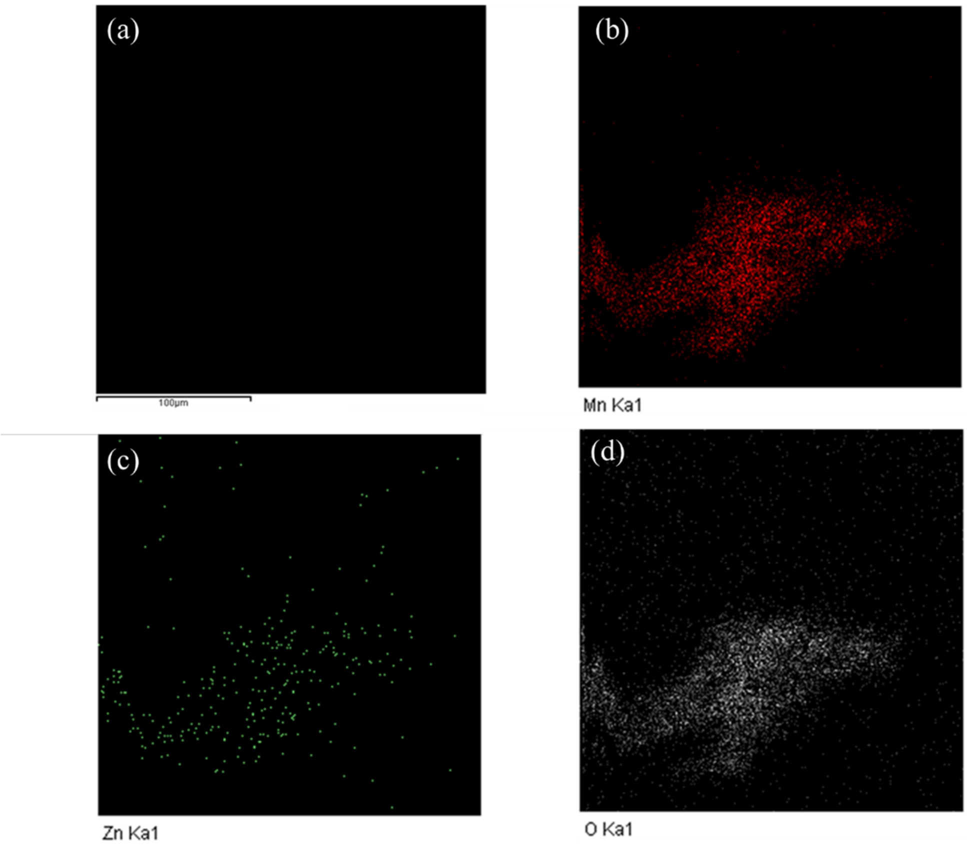

Figure S2. Images of energy-dispersion X-ray spectroscopy (EDX) (**a**) and element mapping of Mn (**b**), Zn (**c**) and O (**d**), respectively.
